# Catestatin as a Biomarker of Cardiovascular Diseases: A Clinical Perspective

**DOI:** 10.3390/biomedicines9121757

**Published:** 2021-11-25

**Authors:** Josko Bozic, Marko Kumric, Tina Ticinovic Kurir, Hrvoje Urlic, Dinko Martinovic, Marino Vilovic, Nada Tomasovic Mrcela, Josip A. Borovac

**Affiliations:** 1 Department of Pathophysiology, University of Split School of Medicine, 21000 Split, Croatia; marko.kumric@mefst.hr (M.K.); tticinov@mefst.hr (T.T.K.); dinko.martinovic@mefst.hr (D.M.); marino.vilovic@mefst.hr (M.V.); 2 Department of Endocrinology, Diabetes and Metabolic Diseases, University Hospital of Split, 21000 Split, Croatia; 3 Department of Cardiovascular Diseases, Clinic Magdalena, 49217 Krapinske Toplice, Croatia; hrvoje.urlic@gmail.com; 4 Andrija Štampar Teaching Institute of Public Health, Referral Center for Health Care of the Elderly of the Ministry of Health of the Republic of Croatia, Department of Public Health Gerontology, 10000 Zagreb, Croatia; nada.tomasovic@du.t-com.hr; 5 Department of Health Studies, University of Split, 21000 Split, Croatia; jborovac@mefst.hr; 6 Department of Cardiology, University Hospital of Split, 21000 Split, Croatia

**Keywords:** catestatin, chromogranin A, biomarker, cardiovascular disease, heart failure, sympathetic nervous system, catecholamine

## Abstract

Accounting for almost one-third of the global mortality, cardiovascular diseases (CVDs) represent a major global health issue. Emerging data suggest that most of the well-established mechanistic explanations regarding the cardiovascular pathophysiology are flawed, and cannot fully explain the progression and long-term effects of these diseases. On the other hand, dysregulation of the sympathetic nervous system (SNS) has emerged as an important player in the pathophysiology of CVDs. Even though upregulated SNS activity is an essential compensatory response to various stress conditions, in the long term, it becomes a major contributor to both cardiac dysfunction and vascular damage. Despite the fact that the importance of SNS hyperactivity in the setting of CVDs has been well-appreciated, its exact quantification and clinical application in either diagnostics or therapy of CVDs is still out of reach. Nevertheless, in recent years a number of novel laboratory biomarkers implicated in the pathophysiology of SNS activation have been explored. Specifically, in this review, we aimed to discuss the role of catestatin, a potent physiological inhibitor of catecholamine spillover that offers cardioprotective effects. Limited data indicate that catestatin could also be a reliable indirect marker of SNS activity and it is likely that high CST levels reflect advanced CV disease burden. Consequently, large-scale studies are required to validate these observations in the upcoming future.

## 1. Introduction

Accounting for almost a third of the global mortality, cardiovascular diseases (CVDs), an umbrella term for several linked pathologies of the CV system, still represent a major global health issue [1]. Despite the accumulation of large amount of data from both basic and clinical research, there are still many mechanisms underlying CV pathologies that are yet to be elucidated. Namely, the emerging data suggest that most of the well-established mechanistic explanations regarding the CV pathophysiology are flawed, and cannot fully explain the progression and long-term effects of these diseases [2]. On the other hand, neurohormonal derangements, including the dysregulation of the renin-angiotensin-aldosterone system (RAAS) and the sympathetic nervous system (SNS), have emerged as important players in the pathophysiology of CVDs, notably in regard to heart failure (HF) [3].

SNS activation is one of the fundamental responses of the human body to stress, which acts by inducing and/or modifying a wide spectrum of potent hemodynamic effects, including positive inotropic, lusitropic, chronotropic, and dromotropic effects on the heart, as well as centralization of the blood flow via vasoconstriction [4]. The degree of SNS activation, with consequent effects on cardiac function and peripheral circulation, is regulated by complex integration of the autonomic CV reflexes [5]. Even though upregulated SNS activity is an essential compensatory response to various stress conditions, such as hypovolemia, hypoxia, or hypoglycemia, in the long-term, it becomes a major contributor to both cardiac dysfunction and vascular damage [6].

Despite the fact that the importance of SNS hyperactivity in the setting of CVDs has been appreciated, its exact quantification and clinical application in either diagnostics or therapy of CVDs is still out of reach. Nevertheless, in the recent years, a number of novel laboratory biomarkers implicated in the pathophysiology of SNS activation have been explored [7]. Specifically, in this review, we aimed to discuss the role of catestatin, a product of the precursor chromogranin A (ChgA), which modulates the spillover of catecholamines, in the setting of different CV pathologies and to debate whether catestatin could serve as a biomarker and/or therapeutic target for these pathologies in the upcoming future [8]. Although initially identified as a physiological inhibitor of catecholamine secretion, catestatin has recently emerged as a potent pleiotropic peptide that leads to a reduction in arterial blood pressure, and positively regulates baroreflex sensitivity and heart rate variability, providing cardioprotection.

## 2. Overview of the Physiological Effects of Catestatin

Catestatin, a 21 amino acid long product of precursor hormone ChgA, was first isolated in 1997 by Mahata et al. [8]. ChgA is encoded by the human CgA gene (CHGA), located on the human chromosome 14, from which a 457 amino acid long protein is translated [9]. Along its length, ChgA contains 8–10 dibasic residues that act as sites of proteolytic cleavage, enabling creation of several peptides with distinct physiological functions [10]. These peptides include vasostatin (ChgA^1–76^), pancreastatin (ChgA^250–301^), catestatin (ChgA^352–372^), and serpinin (ChgA^411–436^) [8]. Apart from being secreted from neuroendocrine tissues and nerve endings, ChgA is widely distributed in the secretory granules of the skin, sensory organs, and myocardium, and novel data suggest that ChgA is preferentially processed in the bone marrow of multiple myeloma patients [11,12]. ChgA, alongside other soluble secretory proteins that are co-stored and co-released with catecholamines in vesicles, exerts an important modulatory role in the adrenergic system [13]. In addition, accumulating evidence suggests that ChgA in humans is also produced in the myocardium, where it causes negative inotropic and lusitropic effects, thus providing evidence for the neuroendocrine regulation of cardiac function by ChgA, and where it is processed further to catestatin, which has direct effects on the myocardium, as we will further discuss [14,15].

Upon the stimulation of sympathetic axons or chromaffin cells, ChgA is released via exocytosis and undergoes extracellular post-translational proteolytic processing via pro-protein convertases, resulting in the release of several bioactive polypeptides, including catestatin. Subsequently, catestatin reversibly and noncompetitively antagonizes neuronal nicotinic cholinergic receptors (nAChR) by occluding the extracellular opening of the channel pore [16]. As nAChR stimulation brings the exocytosis of chromaffin granules containing multiple neurohormones, neuropeptides, and catecholamines, by antagonizing nAChR, catestatin negatively regulates catecholamine release in an autocrine fashion [17]. Specifically, alongside catecholamines, catestatin inhibits the exocytotic release of NPY, adenosine triphosphate, chromogranins, and others; thus, it has emerged as a potent regulator of neuropeptide transmission in the sympathochromaffin system [17]. Notably, by binding to nAChR, catestatin has been shown to inhibit the desensitization of nAChR-mediated catecholamine release, thus facilitating sustained catecholamine release in an organism undergoing stress [18,19].

**Figure 1:**
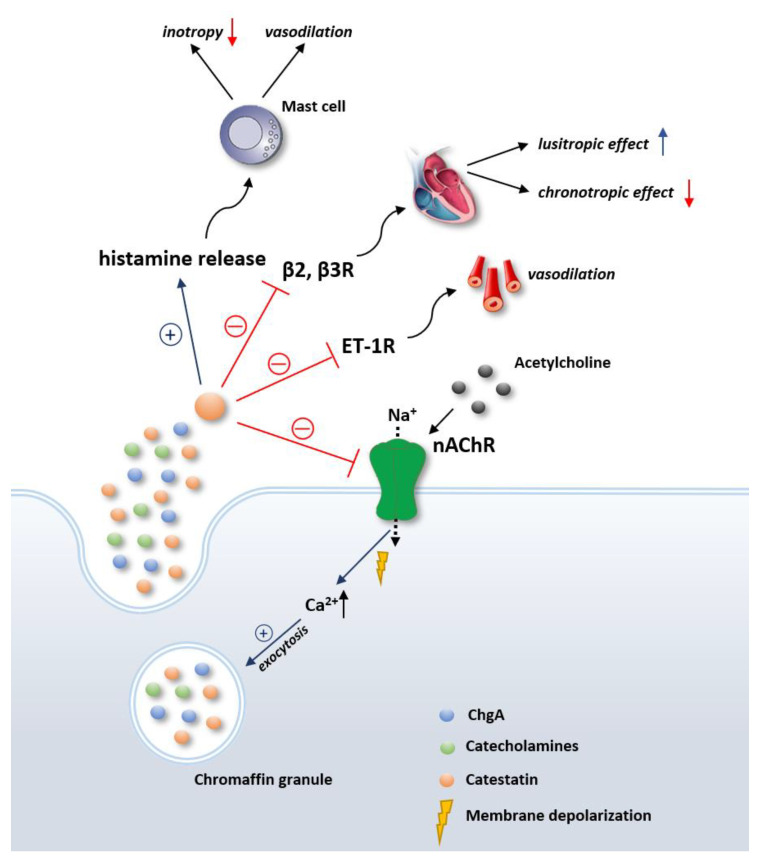
Various receptors through which catestatin exerts its functions. Inhibition of nAchR interferes with membrane depolarization, which then prevents influx of calcium needed for exocytosis of chromaffin granules. Red lines depict inhibition; blue lines indicate stimulation. Abbreviations: ET-1R: endothelin-1 receptor; nAchR: nicotinic acetylcholine receptors; β2, β3R: beta 2 and beta 2 receptor; ChgA: chromogranin A.

Apart from the nAChR, which is by far the most elaborated putative catestatin receptor, catestatin has been shown to bind several other receptors as well (Figure 1). Binding of different receptors, with the consequent activation of various signal transduction pathways, in fact enables catestatin to exhibit a multitude of different functions. Although direct interaction between the histamine H1 receptor and catestatin has not been evaluated yet, animal studies suggest that catestatin may stimulate histamine release via a receptor-independent mechanism, thus displaying a transient positive inotropic effect, followed by a prolonged negative inotropic effect in the heart and vasodepressive activity [17,20,21]. These effects were abolished in the presence of an H1 receptor antagonist, validating the abovementioned hypothesis [22]. Furthermore, it was demonstrated in preclinical studies that catestatin can bind β2- and β3-, but not β1-adrenergic receptors, producing negative inotropic and positive lusitropic effects via the NO-dependent pathway [21]. In line with this, a recent study showed that catestatin effectively blunted the effects of norepinephrine and other mitogenic signals on β1- and β2-adrenergic receptors, thus providing evidence that catestatin has a direct modulatory effect on adrenergic transmission at the level of adrenergic receptors. Finally, some of the vasodilatory role of catestatin could be due to endothelin-1 (ET-1) receptor blockage, as catestatin vasodilated ET-1-mediated coronary vasoconstriction in rats, and as the abovementioned effects were abolished in the case of pre-treatment with ET-1 receptor blocker in a frog heart model [23,24].

The data suggest that various single nucleotide polymorphisms (SNPs) present in the catestatin-expressing region of the *CHGA* gene in different human populations lead to different variants of catestatin [25]. Each of these variants seems to exert different potency in performing catestatin-mediated activity. Although the mechanistic basis of this observation remains elusive, it is possible that the differences in structure among different variants of catestatin may be the reason. Specifically, structural differences among the peptide variants might alter their physical interactions with various downstream molecules involved in the catestatin-mediated signaling pathways [26]. For instance, it has been shown that effectiveness in the inhibition of catecholamine release depends on the extent to which the catestatin variant can occlude the cationic pore of the nAChR [26,27,28]. So far, only a limited amount of populational studies have explored the effects of different catestatin variants on indices of CV health. Hence, as these variants might have potential clinical applications, further studies are needed to explore their putative associations with clinical endpoints, such as mortality, major adverse cardiovascular events (MACE), and the incidence of CV pathologies, in large populations.

## 3. Catestatin in Cardiovascular System Regulation

A myriad of studies suggest that catestatin is implicated in CV system regulation both by central and peripheral mechanisms. In the central nervous system (CNS), depending on the region of medulla where catestatin is expressed, catestatin exerts both sympathoexcitatory and procholinergic effects. For instance, the injection of catestatin into the rostral ventrolateral medulla (RVLM), a key site for blood pressure control in the brain stem, resulted in the activation of sympathoexcitatory bulbospinal neurons, increased barosensitivity, and the attenuation of chemosensitivity and the somatosympathetic reflex with the consequent elevation of blood pressure [29]. Conversely, the injection of catestatin into the caudal ventrolateral medulla (CVLM) of rats, composed of GABAergic interneurons that inhibit RVLM neurons, resulted in decreased sympathetic barosensitivity and the attenuation of the peripheral chemoreflex with consequent hypotension [30]. Similarly, the injection of catestatin into the central amygdala, which consists of inhibitory neurons of the RVLM as well, resulted in decreased blood pressure [31]. Altogether, these findings indicate that catestatin acts as an excitatory peptide in the CNS, as opposed to its inhibitory action in the periphery, and suggests its important role in central cardiorespiratory control. In line with this, catestatin plays a role in the CV system by restoring the sensitivity of high-pressure baroreceptors. Specifically, it has been shown that catestatin attenuated both the reflex tachycardia caused by sodium nitroprusside-induced hypotension and the reflex bradycardia caused by phenylephrine-induced hypertension in a *Chga*-KO mouse model [32]. Another regulatory function of catestatin in the CV system is seen by the improvement of heart rate variability, a reliable indicator of interplay between the SNS and the parasympathetic nervous system. Namely, it has been shown in hypertensive and hyperadrenergic *Chga*-KO mice that HRV indices were normalized upon treatment with catestatin [33]. In the clinical setting, Rao et al. demonstrated corroboration with the abovementioned preclinical data, as Gly364Ser variants displayed increased cardiac parasympathetic index and lower cardiac sympathetic index, implying that this variant of catestatin caused profound changes in human autonomic activity, both parasympathetic and sympathetic [34].

It has so far been well-established that inflammation plays a prominent role in the development of CVD. According to the available evidence, inflammation may also be one of the effector arms through which catestatin contributes to the regulation of the CV system. Namely, catestatin has been shown to modulate multiple immune cell functions. In mast cells, it has been reported that catestatin stimulates the release of histamine with even higher potency and efficacy than the wasp venom peptide mastoparan [35]. Importantly, it seems that histamine-induced vasodilation is largely responsible for the vasodilatory effects of catestatin, as catestatin-induced vasodilation remained unaffected after adrenergic (both α and β) blockade. In line with this, Fung et al. administered catestatin via the dorsal hand vein after pharmacologic venoconstriction with phenylephrine and demonstrated dose-dependent vasodilation [36]. These findings may, in part, explain the hypotensive effects of catestatin. Moreover, Zhang et al. demonstrated that catestatin was able to penetrate into neutrophils and stimulate release of several potent proteins, including neutrophil gelatinase associated lipocalin (NGAL) [37]. The latter may be important, as evidence suggests that NGAL can improve post-infarctional cardiac healing and outcomes by steering macrophage polarization towards the reparative phenotype, thus implying a potential role for catestatin in this setting [38].

In summary, the cardiovascular effects of catestatin are biologically heterogeneous and pleiotropic, encompassing suppression of beta-adrenergic activation through which catestatin acts in a negative inotropic, lusitropic, and chronotropic manner, direct vasodilatory effects on the periphery by activation of mast cells, stimulation of angiogenesis and proliferation of vascular smooth muscle cells, decrease in thrombogenicity of endothelial cells, and suppression of atherosclerosis and inflammation. Finally, catestatin exerts multiple cardioprotective effects, such as abrogating cardiomyocyte ischemia-reperfusion (I/R) injury and the attenuation of adverse cardiac remodeling and hypertrophy [39,40,41].

Of note, Penna et al. were the first to demonstrate direct cardioprotective effects of catestatin on adult rat cardiomyocytes exposed to I/R injury; catestatin increased the cell viability rate by 65%, improved diastolic left ventricular pressure, and significantly improved post-ischemic recovery of the left ventricle [42]. Bassino and colleagues were the first to demonstrate the direct protective effects on rat cardiomyocytes by stimulating phosphoinositide 3-kinase, protein kinase B (PI3K/Akt), glycogen synthase kinase-3 beta (GSK3β) pathway, which led to preservation of mitochondrial membrane potential [43]. Similar findings were previously corroborated [44]. Furthermore, anti-apoptotic effects of catestatin were also achieved by activating the type 2 muscarinic acetylcholine receptor in cardiomyocytes that are exposed to I/R injury, as this led to the upregulation of the activated extracellular signal-regulated kinases 1/2 (ERK1/2) and PI3K/Akt pathways that inhibited endoplasmic reticulum (ER)-induced cell apoptosis [45]. Moreover, Chu et al. demonstrated that the salvage of oxidative stress-induced apoptotic cardiomyocytes in ischemia-reperfusion injury by catestatin might involve the activation of the β2 receptor and the regulation of signaling through the reperfusion injury salvage kinase (RISK) pathway [40]. Similarly, the anti-apoptotic effects of CST in the setting of experimentally induced ischemia-reperfusion injury improved cardiomyocyte hemodynamics, reduced infarct size, and preserved antioxidant effects provided by superoxide dismutase and glutathione peroxidase in the same study. Finally, a genotypic study confirmed that amino acid substitution variants within the catestatin region differentially regulated infarct size in the rat model of I/R injury, showing that variations in the catestatin peptide impacted on the magnitude of reperfusion injury [46].

## 4. Role of Catestatin in Various Cardiovascular Disorders

Being recognized as a potent endogenous inhibitor of catecholamine secretion, it was initially hypothesized that catestatin may be implicated in the pathophysiology of catecholamine-mediated hypertension. The first line of evidence to support these notions was established in a model of *Chga*-KO mice [47]. These mice had a hypertensive and hyperadrenergic phenotype that was normalized using exogenous catestatin. Subsequently, it has been demonstrated that catestatin provides direct vasodilative effects and that the naturally occurring human CST-Gly364Ser variant was associated with an increased risk of hypertension, with age-, sex-, and BMI-adjusted odds ratio of 1.469 (95%CI 1.087–1.984) [48]. Moreover, 364Ser allele carriers displayed increased systolic (≈8 mmHg) and diastolic (≈6 mmHg) blood pressure. The molecular basis of these observations may lie in a distortion of the backbone of the peptide caused by this genetic variant [49]. Subsequently, structural differences such as this one might alter physical interactions with various downstream molecules involved in the catestatin-mediated signaling cascades, thus resulting in changed catecholamine inhibition. Furthermore, plasma levels of the catestatin precursor ChgA were two-fold higher in patients with essential hypertension when compared to normotensive counterparts [50]. Conversely, O’Connor et al. demonstrated that catestatin was diminished early in the course of the development of hypertension, and even in the normotensive offspring of patients with the disease [51]. As catestatin predicts augmented adrenergic pressor responses, the authors suggested that this might represent the mechanism through which diminished catestatin might increase the risk for later development of hypertension. The inverse relationship between ChgA and catestatin in hypertension might be explained by reduced ChgA to catestatin conversion. Specifically, it has been shown that myocardial ChgA to catestatin conversion is impaired because of hyperglycosylation in individuals with HF [52]. Importantly, ChgA hyperglycosylation seems to be independent of glucose plasma levels, but rather a result of increased intracardiac *O*-GlcNAcylation, a post-translational protein modification that represents an important intracellular signaling system in the failing heart [53]. Accordingly, the abovementioned mechanism of ChgA hyperglycosylation (leading to reduced catestatin levels) might operate in essential hypertension as well. Recent preclinical data corroborate that catestatin acts as an autocrine attenuator of cardiac inflammation in hypertension by reducing macrophage inflammation in the heart as catestatin-KO mice did not only develop a hypertensive phenotype, but also showed marked left ventricular hypertrophy, macrophage infiltration of the heart and adrenal glands, as well as elevated levels of proinflammatory cytokines and catecholamines [54]. This work was the first to show that the immunosuppression of macrophages is a driving mechanism behind the cardioprotective effects of catestatin.

Apart from hypertension, studies suggest that catestatin may also be implicated in the pathophysiology of malignant arrythmias. Namely, the chronic administration of exogenous catestatin reduced the incidence of experimentally induced ventricular arrhythmias in a rat model of myocardial infarction (MI) [55]. Similarly, in the rat model of hyperadrenergic hypertension, rats with ablated ChgA gene showed significantly longer QT interval and QRS time–voltage, accompanied by increased resting heart rate and QT variability, thus demonstrating that arrhythmogenic ventricular assault develops with the condition of low circulating catestatin levels [56]. The abovementioned data from preclinical studies were subsequently validated in the clinical setting, as elevated catestatin levels were an independent predictor of complicated malignant arrhythmias among patients with acute MI [57]. Although this might seem counterintuitive at first, elevated catestatin levels could reflect its compensatory response to increased SNS activity and excess catecholamine discharge. Therefore, this could be an attempt to restore autonomic balance, and circulating catestatin levels may reflect biological catecholamine turnover and the degree of sympathetic activity, as catestatin co-localizes and is co-released with catecholamines and other neuropeptides. It is important to address that the putative cardioprotective effects of catestatin are likely overpowered by excessive sympathetic discharge in the conditions of deranged CV homeostasis, such as acute MI or decompensated HF, despite relatively high circulating levels of catestatin.

**Figure 2:**
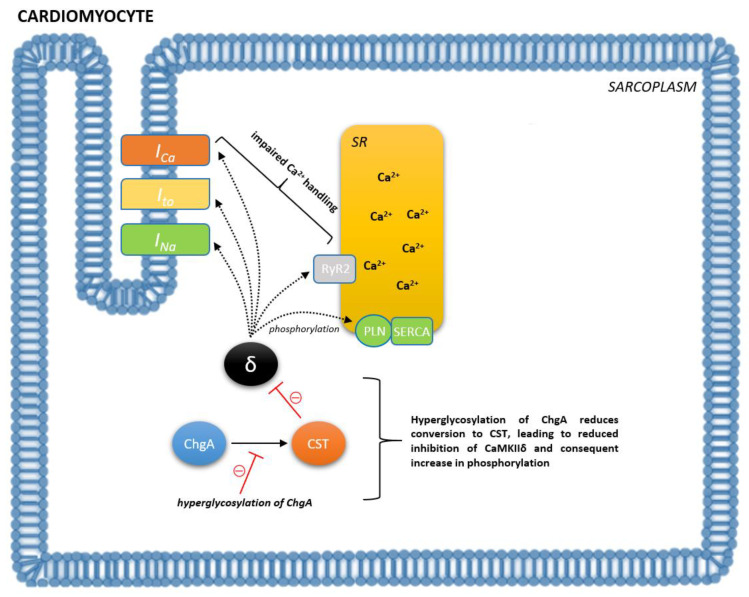
Putative mechanism by which catestatin contributes to pathophysiology of heart failure (HF). In the setting of HF, hyperglycosylated ChgA cannot be sufficiently cleaved by CST, which leads to reduced inhibition of CaMKIIδ and CaMKIIδ-mediated phosphorylation of cardiac proteins. As a result of increased phosphorylation, calcium handling in cardiomyocytes becomes impaired and dampens cardiac function. Abbreviations: ChgA: chromogranin A; CST: catestatin; CaMKIIδ (δ): the multifunctional Ca^2+^/calmodulin-dependent protein kinase II delta; SR: sarcoplasmic reticulum; RYR2: ryanodine receptor 2; SERCA: sarco/endoplasmic reticulum Ca^2+^-ATPase; PLN: phospholamban.

Increased sympathetic nerve activity has been recently recognized as a major contributor to the pathophysiology of HF. In fact, the SNS-induced rapid increase in the effective circulating volume, resulting in the mobilization of fluid from the splanchnic bed, is presumed to be the dominant driving force behind increased central venous pressure and congestion encountered during HF decompensation [58,59]. Therefore, it is not surprising that ChgA and its fragments are implicated in the pathophysiology underlying HF. As previously mentioned, it has been demonstrated that myocardial ChgA to catestatin conversion is impaired as a result of hyperglycosylation in HF individuals, thus resulting in an increase in phosphorylation of phospholamban and ryanodine receptor 2 via the reduced inhibition of CaMKIIδ (Figure 2) [52]. Moreover, an intramyocardial production of ChgA is established in humans and was associated with negative inotropic and lusitropic effects on the mammalian heart, thus providing evidence for the neuroendocrine regulation of cardiac function by ChgA [60]. It is plausible that the observed effects are a result of ChgA fragments, notably catestatin and vasostatin, as both of those have demonstrated these functions in the myocardium, respectively. Importantly, immunohistochemical biopsy studies showed that ChgA is co-localized with BNP in the dilated and hypertrophic left ventricle, with ChgA levels correlating with end-diastolic left ventricular pressures [60]. Altogether, these results provide support for the direct functional role of ChgA and its fragments in the setting of HF. In line with the pathophysiological background, clinical studies showed that levels of ChgA were elevated in the peripheral blood of patients with chronic HF (5–10-fold increase), dilated cardiomyopathy/hypertrophic cardiomyopathy (2.5-fold increase), and MI patients with symptoms of HF (0.61 vs. 0.49 nM), correlating with both mortality and poor outcomes [14,61,62,63,64,65,66]. However, the largest trial evaluating the prognostic value of ChgA in chronic HF yielded disappointing results, as plasma ChgA levels in patients with chronic HF did not provide any incremental prognostic information over that obtained from physical examination, routine biochemical analysis, and standard HF biomarkers [65]. On the contrary, Jansson et al. demonstrated that ChgA measured on day 1 was an independent predictor of long-term mortality and HF hospitalizations for patients presenting with ACS, and provided incremental prognostic information to conventional CV risk markers [66].

As for catestatin, a limited number of studies examined the role of CST in HF and investigated its prognostic role in this setting. Zhu et al. showed that catestatin levels gradually decreased from stage A to C of HF, while there was no difference between stage A and B in terms of catestatin levels with a cut-off value of 19.73 ng/mL, yielding 90% sensitivity and 50.9% specificity for the detection of B stage of HF [67]. This finding may be of clinical relevance, since stage B presumes structural cardiac disorder but without symptoms, whereas stage A assumes patients are at a high risk of developing HF without functional or structural heart disorders. Therefore, the abovementioned study implied that decreased catestatin levels may point to structural heart disease among asymptomatic patients. Furthermore, in a study by Liu et al., catestatin was higher in patients with HF compared to control subjects, and it was positively correlated with NYHA class [68]. Moreover, ischemic etiology of HF and NYHA class predicted plasma catestatin levels, whilst BNP provided a better area under the curve (AUC) value than catestatin for the detection of moderate to severe HF (0.831 vs. 0.626) without improving diagnostic accuracy when catestatin was added to BNP. In the recently conducted CATSTAT-HF study, patients with a higher NYHA functional class had higher serum catestatin levels in comparison to the counterparts with lower NYHA in a population of acutely decompensated HF patients [69]. In line with this, catestatin levels were higher in the subgroup of patients with ischemic etiology of HF (history of MI), likely reflecting an augmented SNS neurohumoral activation in patients with this etiopathogenetic mechanism of HF. Accordingly, Zhu et al. demonstrated that catestatin might be a novel marker reflecting LV remodeling in the myocardium following acute MI, as a positive association between catestatin concentrations at day 3 post-MI and LV remodeling indices was established and as patients with higher catestatin levels developed worse ventricular function during the follow-up period [69]. In addition, single-point catestatin levels were effective in the prediction of LVEDD change, whereas concurrently increasing catestatin and NT-proBNP levels were predictive of the highest risk of LV remodeling. The CATSTAT-HF study also demonstrated that catestatin serum levels independently correlated with NYHA class, high-sensitivity cardiac troponin I, and heart rate at both admission and rest, waist-to-hip ratio, HbA1c, LDL cholesterol, and non-HDL cholesterol. Although the CATSTAT-HF showed no difference in catestatin levels with respect to HF phenotypes stratified by the degree of systolic function, higher catestatin levels exhibited a firm association with favorable echocardiographic profile, as they were positively correlated with smaller LV volumes and dimensions, decreased left ventricular mass and smaller dimensions of the left atrium. The above- findings were in line with the postulated local cardioprotective effects of catestatin [70,71,72]. Finally, as demonstrated by Peng et al., plasma catestatin levels higher than the upper tertile cut-off value of 1.096 ng/mL emerged as an independent risk factor for all-cause death (HR: 1.84, 95%CI: 1.02–3.32) and cardiac death (HR: 2.41, 95%CI: 1.26–4.62) in the multivariate Cox regression analysis [73]. Moreover, in patients who exhibited both high catestatin and natriuretic peptide levels during hospitalization, the risk of all-cause death increased three-fold and the risk of cardiac death increased four-fold. Subsequently, Wołowiec et al. aimed to assess the usefulness of plasma concentrations of catestatin as a predictor of a composite endpoint: unplanned hospitalization and death for all causes in 52 Caucasian patients with HFrEF in the midterm follow-up (24 months) [74]. The authors concluded that catestatin levels measured before and after physical exertion were a valuable prognostic parameter in predicting death from all causes and unplanned hospitalization in this group of patients, with increased importance of catestatin in the long-term follow-up. However, the marked methodological feature of this study should be highlighted. Of note, this study included stable HFrEF patients and CST levels were not measured in the decompensated state while none of patients received intravenous diuretic or inotropic therapy; thus, most were treated on an outpatient basis.

In line with this, Izci and colleagues demonstrated that catestatin levels were higher in patients with acute pulmonary embolism (PE) than in the control group [75]. Moreover, the authors demonstrated that catestatin levels correlated with simplified Pulmonary Embolism Severity Index (sPESI), an algorithm utilized commonly for estimating 30-day mortality in patients diagnosed with non-high-risk PE [76]. In addition, catestatin blood levels correlated with indices of right ventricular dysfunction. Overall, these results imply that catestatin is involved in the pathophysiology of right-sided HF, as it likely interacts with sympathetic discharge in acute PE and the severity of this discharge likely closely parallels catestatin levels.

Apart from the well-established role of catestatin in CVD, the data indicate that catestatin is linked to inflammatory and metabolic syndrome diseases and can be a novel regulator of insulin and lipid levels [77]. In an obese mouse model it was demonstrated that catestatin infusion leads to body weight reduction mediated by the inhibition of alpha2-adrenergic receptor signaling and the enhancement of leptin receptor signaling [78]. On the other hand, systemic catestatin-KO mice fed with normal chow diet (NCD) consumed more food, gained weight, and displayed elevated insulin and blood glucose levels, whereas supplementation with catestatin normalized these parameters. In the wild-type mice fed with NCD, catestatin supplementation affected neither glucose tolerance nor insulin sensitivity, whilst it improved these parameters in both obese wild-type and catestatin-KO mice [79]. In accordance, Dasgupta et al. showed that catestatin may ameliorate obesity-induced hepatic insulin resistance by reducing inflammation, inhibiting proinflammatory macrophage infiltration, and reducing ER stress [80]. In obese pediatric subjects, our study showed that plasma catestatin levels were significantly lower in obese subjects in comparison to non-obese counterparts (10.57 ± 5.13 vs. 13.49 ± 6.18 ng/mL, *p* < 0.05). Furthermore, when dividing obese subjects in two groups according to the presence of metabolic syndrome, catestatin was significantly lower in the subgroup of obese children with concomitant metabolic syndrome (8.52 ± 3.89, *p* < 0.05), showing that the evidence in mice models regarding the role of catestatin in metabolic disorders translated to the clinical setting [81,82].

## 5. Biomarker Potential of Catestatin and Future Directions

Before any further discussion with respect to the role of catestatin as a CV biomarker, we need to reflect on the problematic nature of biomarker establishment. Namely, each newly developed biomarker has to be assessed by its appropriateness to answer fundamental questions in order to determine its clinical relevance: in which group/subgroup of patients should the marker be measured; at which point in time should the measurement be employed; does the biomarker provide additional information beyond existing biomarkers; and finally, whether the biomarker yields auspicious features in a cost-effective analysis.

It is obvious from the aforementioned studies that catestatin is implicated in the pathophysiology of multiple cardiovascular disorders, most notably in HF, arterial hypertension, I/R injury, and malignant arrythmias in ACS. Nonetheless, the putative role of catestatin as a biomarker in these pathologies is still very speculative. The clinical setting in which application of catestatin measurement may be most promising is heart failure. Of note, based on the presented data, it is plausible that catestatin may be a reliable indirect marker of SNS activity and that high catestatin levels could reflect advanced disease burden and high sympathoexcitatory profile among HF patients. Furthermore, a favorable fact is that catestatin seems to provide incremental prognostic information to natriuretic peptides in terms of HF mortality and could aid thus aid in the risk stratification of chronic HF in both stable and decompensated settings. However, such findings are based on the limited evidence; therefore, further large-scale studies are required to validate these findings and clarify the role of catestatin for prognostic purposes in HF. In line with this, catestatin emerged as a useful biomarker for identifying individuals at high risk of in-hospital death among patients admitted with acute worsening of HF. It is doubtful that catestatin will replace natriuretic peptides in HF, but studies suggest that a multimarker approach including natriuretic peptides, sST2, catestatin and other molecules may improve risk stratification in HF patients. Importantly, HF patients with elevated catestatin levels may also be appropriate candidates for the introduction or up-titration of sympatholytic agents and require more stringent monitoring. However, we are still far from catestatin-tailored therapies, as the effects of neurohumoral antagonists on circulating catestatin levels are yet to be elucidated.

Of equal importance, the application of catestatin not only as a diagnostic or prognostic biomarker, but as an in vivo therapeutic agent itself is an attractive concept that should be explored in the future. This approach should be embraced and investigated in humans as a solid body of preclinical evidence is available demonstrating the favorable cardioprotective and hemodynamic effects of exogenously administered catestatin. Finally, albeit not widely used, catestatin is relatively cheap and easily available, but to ascertain the economical aspect of catestatin in HF, a cost-benefit analysis should be performed in future studies. Nevertheless, it is very important to acknowledge that most of the data concerning catestatin is based on animal models and thus may not exactly reflect processes occurring in the human body.

## 6. Conclusions

In conclusion, through inhibition of catecholamine spillover and by exerting a myriad of cardioprotective effects, catestatin has found its place in the complex pathophysiology of cardiovascular diseases. In short, elevated levels of catestatin seem to reflect SNS hyperactivity and excess catecholamine discharge. Given that catestatin provides an incremental value in risk stratification of patients with chronic HF, it is plausible that catestatin may become a part of the prognostic multimarker panel used in the management of this clinical syndrome. In addition, elevated catestatin plasma levels may guide clinicians towards more stringent monitoring and aggressive treatment of acutely decompensated HF patients or HF outpatients. Furthermore, catestatin may also aid in the prediction of malignant arrhythmias in acute MI patients. Nevertheless, to establish the putative role of catestatin as a biomarker, large-scale follow-up studies with cost-benefit analyses are warranted. Finally, the use of catestatin, not only as a biomarker but as a therapeutic agent in various CV disorders, warrants further exploration in the future.

## Data Availability

Not applicable.
